# 2-Methyl­pyridine–urea (1/1)

**DOI:** 10.1107/S1600536812002164

**Published:** 2012-01-21

**Authors:** Jamshid Ashurov, Bakhtiyar Ibragimov, Samat Talipov

**Affiliations:** a Institute of Bioorganic Chemistry, Academy of Sciences of Uzbekistan, H. Abdullaev Str. 83, Tashkent 100125, Uzbekistan

## Abstract

In the crystal structure of the title compound, C_6_H_7_N·CH_4_N_2_O, the 2-methyl­pyridine and urea mol­ecules are linked *via* N—H⋯O and N—H⋯N hydrogen bonds, forming ribbons extending along the *a* axis. The dihedral angle between the 2-methyl­pyridine and urea mean planes is 89.09 (9)°. The methyl group shows rotational disorder wherein the H atoms are located over two sets of sites with equal occupancies.

## Related literature

For crystal structures of urea inclusion compounds, see: Izotova *et al.* (2008 ▶); Chadwick *et al.* (2009 ▶).
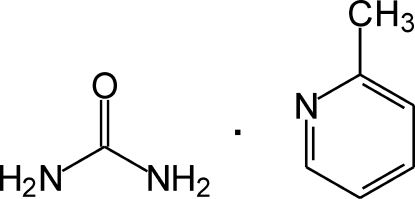


## Experimental

### 

#### Crystal data

C_6_H_7_N·CH_4_N_2_O
*M* *_r_* = 153.19
Orthorhombic, 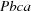

*a* = 7.471 (5) Å
*b* = 14.916 (5) Å
*c* = 15.338 (5) Å
*V* = 1709.2 (14) Å^3^
*Z* = 8
Cu *K*α radiation
μ = 0.68 mm^−1^
*T* = 293 K
0.36 × 0.30 × 0.22 mm

#### Data collection

Oxford Diffraction Xcalibur Ruby diffractometer
Absorption correction: multi-scan (*CrysAlis PRO*; Oxford Diffraction, 2007 ▶) *T*_min_ = 0.337, *T*_max_ = 1.000
5969 measured reflections
1756 independent reflections
1011 reflections with *I* > 2σ(*I*)
*R*_int_ = 0.049

#### Refinement

*R*[*F* ^2^ > 2σ(*F* ^2^)] = 0.056
*wR*(*F* ^2^) = 0.167
*S* = 0.97
1756 reflections
112 parameters
H atoms treated by a mixture of independent and constrained refinement
Δρ_max_ = 0.16 e Å^−3^
Δρ_min_ = −0.20 e Å^−3^

### 

Data collection: *CrysAlis PRO* (Oxford Diffraction, 2007 ▶); cell refinement: *CrysAlis PRO*; data reduction: *CrysAlis PRO*; program(s) used to solve structure: *SHELXS97* (Sheldrick, 2008 ▶); program(s) used to refine structure: *SHELXL97* (Sheldrick, 2008 ▶); molecular graphics: *XP* in *SHELXTL* (Sheldrick, 2008 ▶); software used to prepare material for publication: *SHELXL97*.

## Supplementary Material

Crystal structure: contains datablock(s) I, global. DOI: 10.1107/S1600536812002164/pv2503sup1.cif

Structure factors: contains datablock(s) I. DOI: 10.1107/S1600536812002164/pv2503Isup2.hkl

Supplementary material file. DOI: 10.1107/S1600536812002164/pv2503Isup3.cml

Additional supplementary materials:  crystallographic information; 3D view; checkCIF report

## Figures and Tables

**Table 1:** Hydrogen-bond geometry (Å, °)

*D*—H⋯*A*	*D*—H	H⋯*A*	*D*⋯*A*	*D*—H⋯*A*
N2—H2*B*⋯N1	0.91 (2)	2.26 (2)	3.131 (3)	160 (2)
N2—H2*A*⋯O1^i^	0.84 (2)	2.11 (2)	2.953 (3)	179 (2)
N3—H3*B*⋯N1	0.88 (2)	2.30 (2)	3.137 (3)	161 (2)
N3—H3*A*⋯O1^ii^	0.91 (2)	2.03 (2)	2.938 (3)	177 (2)

Symmetry codes: (i) 
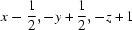
; (ii) 
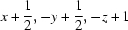
.

**Fig. 1.**
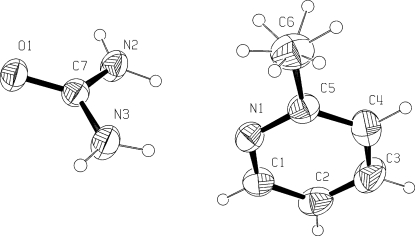
The molecular structure of the title compound with the atom numbering scheme. Displacement ellipsoids are drawn at the 30% probability level. H atoms are presented as small spheres of arbitrary radius.

**Fig. 2.**
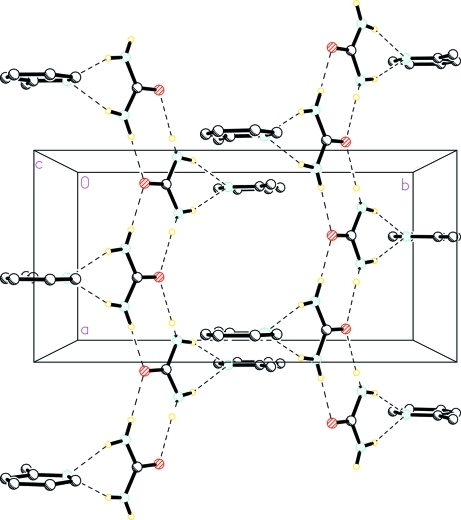
A view of the N—-H···O and N—H···N hydrogen bonds (dotted lines) in the crystal structure of the title compound. H atoms non-participating in hydrogen-bonding were omitted for clarity.

## supplementary crystallographic information

### Comment

The crystal structure of the title compound (Fig. 1) consists of a 2-methylpyridine and a urea molecules. The urea molecules are mutually bonded by pair of N—H···O hydrogen bonds and form infinite chains along the *a*-axis (Fig. 2). The molecules of 2-methylpyridine are connected by its N1-atom to the urea molecules *via* both amino groups resulting in N—H···N type hydrogen bonds. The mean-planes angle between the planes of 2-methylpyridine and urea is 89.09 (9)°. The methyl group of 2-methylpyridine showed rotational disorder with each H-atom located over two positions with occupation factors of 0.50 and 0.50.

The crystal structures of urea inclusion compounds closely related to the title structure have been recently reported (Izotova *et al.*, (2008); Chadwick *et al.*, 2009).

### Experimental

The compound was purchased from Sigma-Aldrich and used as supplied. A single-crystal sample of the 1/1 clathrate was recrystallized from a saturated solution of urea in 2-methylpyridine by isothermal solvent evaporation at room temperature (298 K).

### Refinement

All H atoms bonded to C atoms were placed in calculated positions and refined as riding, with C—H = 0.93–0.96Å with *U*_iso_(H) = 1.2–1.5 *U*_eq_(C). The H atoms of amino group were located from a difference Fourier map and refined freely with *U*_iso_(H) = 1.2*U*_eq_(N).

### Crystal data

**Table:**

C_6_H_7_N·CH_4_N_2_O	*F*(000) = 656
*M**_r_* = 153.19	*D*_x_ = 1.191 Mg m^−^^3^
Orthorhombic, *P**b**c**a*	Cu *K*α radiation, λ = 1.54184 Å
Hall symbol: -P 2ac 2ab	Cell parameters from 1430 reflections
*a* = 7.471 (5) Å	θ = 4.1–75.6°
*b* = 14.916 (5) Å	µ = 0.68 mm^−^^1^
*c* = 15.338 (5) Å	*T* = 293 K
*V* = 1709.2 (14) Å^3^	Block, colourless
*Z* = 8	0.36 × 0.30 × 0.22 mm

### Data collection

**Table:**

Oxford Diffraction Xcalibur Ruby diffractometer	1756 independent reflections
Radiation source: fine-focus sealed tube	1011 reflections with *I* > 2σ(*I*)
graphite	*R*_int_ = 0.049
Detector resolution: 10.2576 pixels mm^-1^	θ_max_ = 76.0°, θ_min_ = 5.8°
ω scans	*h* = −5→9
Absorption correction: multi-scan (CrysAlis PRO; Oxford Diffraction, 2007)	*k* = −18→17
*T*_min_ = 0.337, *T*_max_ = 1.000	*l* = −18→19
5969 measured reflections	

### Refinement

**Table:**

Refinement on *F*^2^	Primary atom site location: structure-invariant direct methods
Least-squares matrix: full	Secondary atom site location: difference Fourier map
*R*[*F*^2^ > 2σ(*F*^2^)] = 0.056	Hydrogen site location: inferred from neighbouring sites
*wR*(*F*^2^) = 0.167	H atoms treated by a mixture of independent and constrained refinement
*S* = 0.97	*w* = 1/[σ^2^(*F*_o_^2^) + (0.0982*P*)^2^] where *P* = (*F*_o_^2^ + 2*F*_c_^2^)/3
1756 reflections	(Δ/σ)_max_ < 0.001
112 parameters	Δρ_max_ = 0.16 e Å^−^^3^
0 restraints	Δρ_min_ = −0.20 e Å^−^^3^

### Special details

**Table:**

Geometry. All e.s.d.'s (except the e.s.d. in the dihedral angle between two l.s. planes) are estimated using the full covariance matrix. The cell e.s.d.'s are taken into account individually in the estimation of e.s.d.'s in distances, angles and torsion angles; correlations between e.s.d.'s in cell parameters are only used when they are defined by crystal symmetry. An approximate (isotropic) treatment of cell e.s.d.'s is used for estimating e.s.d.'s involving l.s. planes.
Refinement. Refinement of *F*^2^ against ALL reflections. The weighted *R*-factor *wR* and goodness of fit *S* are based on *F*^2^, conventional *R*-factors *R* are based on *F*, with *F* set to zero for negative *F*^2^. The threshold expression of *F*^2^ > σ(*F*^2^) is used only for calculating *R*-factors(gt) *etc*. and is not relevant to the choice of reflections for refinement. *R*-factors based on *F*^2^ are statistically about twice as large as those based on *F*, and *R*- factors based on ALL data will be even larger.

### Fractional atomic coordinates and isotropic or equivalent isotropic displacement parameters (Å^2^)

**Table:**

	*x*	*y*	*z*	*U*_iso_*/*U*_eq_	Occ. (<1)
N1	0.1107 (2)	0.45154 (12)	0.27525 (10)	0.0620 (5)	
C1	0.1128 (3)	0.42217 (15)	0.19345 (13)	0.0672 (6)	
H1	0.1138	0.3605	0.1843	0.081*	
C2	0.1137 (3)	0.47652 (19)	0.12219 (13)	0.0763 (7)	
H2	0.1153	0.4528	0.0661	0.092*	
C3	0.1121 (3)	0.5668 (2)	0.13573 (17)	0.0910 (8)	
H3	0.1129	0.6061	0.0887	0.109*	
C4	0.1093 (3)	0.59912 (18)	0.21957 (18)	0.0860 (8)	
H4	0.1076	0.6606	0.2297	0.103*	
C5	0.1089 (3)	0.53974 (15)	0.28912 (13)	0.0645 (6)	
C6	0.1069 (3)	0.5697 (2)	0.38257 (15)	0.0963 (9)	
H6A	0.0145	0.5382	0.4135	0.144*	0.50
H6B	0.0841	0.6329	0.3851	0.144*	0.50
H6C	0.2207	0.5570	0.4089	0.144*	0.50
H6D	0.1984	0.6139	0.3915	0.144*	0.50
H6E	0.1287	0.5191	0.4198	0.144*	0.50
H6F	−0.0078	0.5951	0.3961	0.144*	0.50
O1	0.11116 (17)	0.23145 (9)	0.50993 (9)	0.0637 (4)	
N2	−0.0400 (3)	0.31459 (13)	0.41139 (12)	0.0653 (5)	
H2A	−0.140 (3)	0.3013 (17)	0.4331 (16)	0.078*	
H2B	−0.025 (3)	0.3550 (15)	0.3672 (14)	0.078*	
N3	0.2633 (3)	0.31788 (13)	0.41459 (13)	0.0713 (6)	
H3A	0.370 (3)	0.3003 (18)	0.4372 (16)	0.086*	
H3B	0.248 (3)	0.3551 (16)	0.3712 (14)	0.086*	
C7	0.1115 (3)	0.28588 (12)	0.44860 (12)	0.0526 (5)	

### Atomic displacement parameters (Å^2^)

**Table:**

	*U*^11^	*U*^22^	*U*^33^	*U*^12^	*U*^13^	*U*^23^
N1	0.0673 (11)	0.0681 (10)	0.0505 (9)	−0.0007 (8)	−0.0018 (8)	0.0054 (7)
C1	0.0662 (14)	0.0735 (13)	0.0619 (12)	−0.0010 (11)	−0.0006 (10)	−0.0058 (10)
C2	0.0743 (15)	0.1072 (19)	0.0475 (11)	0.0073 (14)	0.0015 (10)	0.0011 (11)
C3	0.098 (2)	0.108 (2)	0.0674 (14)	0.0061 (16)	0.0033 (14)	0.0344 (14)
C4	0.100 (2)	0.0643 (13)	0.0932 (18)	0.0032 (12)	0.0015 (15)	0.0087 (12)
C5	0.0656 (14)	0.0698 (12)	0.0580 (11)	−0.0027 (11)	0.0015 (10)	−0.0036 (9)
C6	0.107 (2)	0.112 (2)	0.0696 (16)	−0.0082 (16)	0.0021 (14)	−0.0273 (15)
O1	0.0554 (9)	0.0729 (9)	0.0628 (8)	0.0026 (7)	0.0010 (6)	0.0233 (7)
N2	0.0512 (10)	0.0796 (12)	0.0652 (11)	0.0020 (9)	−0.0005 (9)	0.0235 (9)
N3	0.0557 (11)	0.0851 (13)	0.0730 (12)	−0.0003 (10)	0.0030 (9)	0.0315 (10)
C7	0.0527 (11)	0.0552 (9)	0.0498 (9)	0.0006 (9)	0.0006 (9)	0.0040 (8)

### Geometric parameters (Å, °)

**Table:**

N1—C1	1.329 (3)	C6—H6B	0.9600
N1—C5	1.333 (3)	C6—H6C	0.9600
C1—C2	1.361 (3)	C6—H6D	0.9600
C1—H1	0.9300	C6—H6E	0.9600
C2—C3	1.362 (4)	C6—H6F	0.9600
C2—H2	0.9300	O1—C7	1.243 (2)
C3—C4	1.374 (4)	N2—C7	1.338 (3)
C3—H3	0.9300	N2—H2A	0.84 (2)
C4—C5	1.387 (3)	N2—H2B	0.91 (2)
C4—H4	0.9300	N3—C7	1.337 (3)
C5—C6	1.501 (3)	N3—H3A	0.91 (2)
C6—H6A	0.9600	N3—H3B	0.88 (2)
			
C1—N1—C5	118.43 (18)	H6A—C6—H6D	141.1
N1—C1—C2	124.2 (2)	H6B—C6—H6D	56.3
N1—C1—H1	117.9	H6C—C6—H6D	56.3
C2—C1—H1	117.9	C5—C6—H6E	109.5
C1—C2—C3	117.8 (2)	H6A—C6—H6E	56.3
C1—C2—H2	121.1	H6B—C6—H6E	141.1
C3—C2—H2	121.1	H6C—C6—H6E	56.3
C2—C3—C4	119.3 (2)	H6D—C6—H6E	109.5
C2—C3—H3	120.3	C5—C6—H6F	109.5
C4—C3—H3	120.3	H6A—C6—H6F	56.3
C3—C4—C5	119.7 (2)	H6B—C6—H6F	56.3
C3—C4—H4	120.1	H6C—C6—H6F	141.1
C5—C4—H4	120.1	H6D—C6—H6F	109.5
N1—C5—C4	120.5 (2)	H6E—C6—H6F	109.5
N1—C5—C6	116.5 (2)	C7—N2—H2A	120.5 (17)
C4—C5—C6	123.0 (2)	C7—N2—H2B	115.0 (15)
C5—C6—H6A	109.5	H2A—N2—H2B	124 (2)
C5—C6—H6B	109.5	C7—N3—H3A	119.6 (16)
H6A—C6—H6B	109.5	C7—N3—H3B	114.2 (16)
C5—C6—H6C	109.5	H3A—N3—H3B	126 (2)
H6A—C6—H6C	109.5	O1—C7—N3	122.04 (18)
H6B—C6—H6C	109.5	O1—C7—N2	122.02 (19)
C5—C6—H6D	109.5	N3—C7—N2	115.93 (18)
			
C5—N1—C1—C2	0.1 (3)	C1—N1—C5—C4	0.0 (3)
N1—C1—C2—C3	−0.1 (4)	C1—N1—C5—C6	−179.89 (19)
C1—C2—C3—C4	−0.2 (4)	C3—C4—C5—N1	−0.2 (3)
C2—C3—C4—C5	0.3 (4)	C3—C4—C5—C6	179.7 (2)

### Hydrogen-bond geometry (Å, °)

**Table:**

*D*—H···*A*	*D*—H	H···*A*	*D*···*A*	*D*—H···*A*
N2—H2B···N1	0.91 (2)	2.26 (2)	3.131 (3)	160 (2)
N2—H2A···O1^i^	0.84 (2)	2.11 (2)	2.953 (3)	179 (2)
N3—H3B···N1	0.88 (2)	2.30 (2)	3.137 (3)	161 (2)
N3—H3A···O1^ii^	0.91 (2)	2.03 (2)	2.938 (3)	177 (2)

Symmetry codes: (i) *x*−1/2, −*y*+1/2, −*z*+1; (ii) *x*+1/2, −*y*+1/2, −*z*+1.
